# Testing for the lupus anticoagulant: the good, the bad, and the ugly

**DOI:** 10.1016/j.rpth.2024.102385

**Published:** 2024-03-18

**Authors:** Emmanuel J. Favaloro, Leonardo Pasalic, Rita Selby

**Affiliations:** 1 Haematology, Sydney Centres for Thrombosis and Haemostasis, Institute of Clinical Pathology and Medical Research, New South Wales Health Pathology, Westmead Hospital, Westmead, New South Wales, Australia; 2 School of Dentistry and Medical Sciences, Faculty of Science and Health, Charles Sturt University, Wagga Wagga, New South Wales, Australia; 3 School of Medical Sciences, Faculty of Medicine and Health, University of Sydney, Westmead Hospital, Westmead, New South Wales, Australia; 4 Westmead Clinical School, University of Sydney, Westmead, New South Wales, Australia; 5 Department of Laboratory Medicine and Pathobiology, University of Toronto, Toronto, Ontario, Canada; 6 Department of Medicine, University of Toronto, Toronto, Ontario, Canada

**Keywords:** antiphospholipid antibodies, antiphospholipid syndrome, direct oral anticoagulants, DOAC neutralization, lupus anticoagulant

## Abstract

Lupus anticoagulant (LA) represents 1 of the laboratory criteria for classification of patients as having definite antiphospholipid syndrome (APS). The other 2 laboratory criteria are anticardiolipin antibodies and anti–beta2-glycoprotein I antibodies. At least 1 of these antiphospholipid antibody (aPL) tests need to be positive, with evidence of persistence, together with evidence of at least 1 clinical criterion for APS, before a patient can be classified as having definite APS. LA and other aPL assays are also important for diagnosis or exclusion of APS, as well as for risk stratification, with triple-positive patients carrying the greatest risk. Whereas LA is identified through “uncalibrated” clot-based assays, the other aPL assays (anticardiolipin and anti–beta2-glycoprotein I antibodies) represent immunological assays, identified using calibrated solid-phase methods. Because LA is identified using clot-based assays, it is subject to considerable preanalytical and analytical issues that challenge accurate detection or exclusion of LA. In this narrative review, we take a look at the good, the bad, and the ugly of LA testing, primarily focusing on the last 10 years. Although harmonization of LA testing as a result of International Society on Thrombosis and Haemostasis guidance documents and other international activities has led to improvements in LA detection, many challenges remain. In particular, several anticoagulants, especially direct oral anticoagulants and also vitamin K antagonists, given as therapy to treat the pathophysiological consequences of aPL, especially thrombosis, interfere with LA assays and can generate false-positive or false-negative LA findings. Overcoming these diagnostic errors will require a multifaceted approach with clinicians and laboratories working together.

**Figure:**
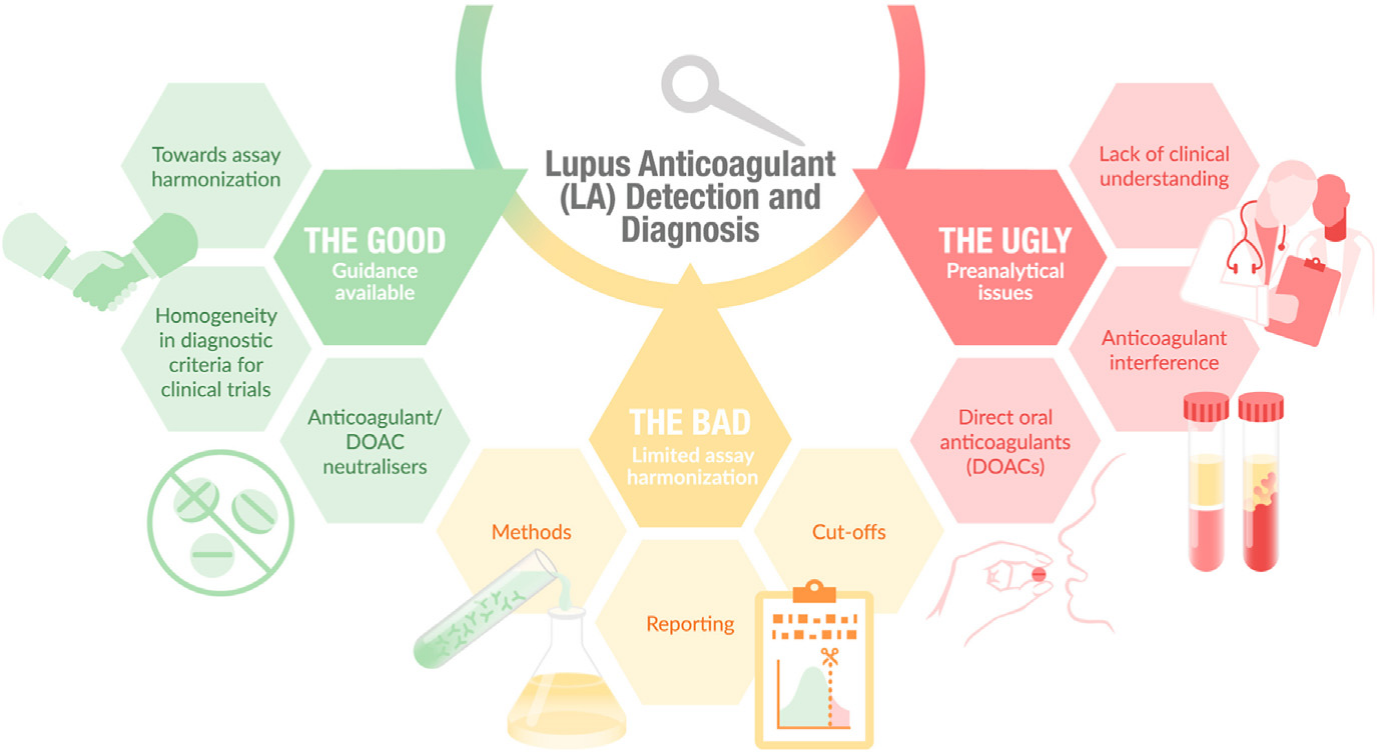

## Essentials

• We summarize the good, bad, and ugly of lupus anticoagulant (LA) testing, focusing on recent years.
• Anticoagulants can interfere with LA assays, generating false-positive/negative findings.
• Guidance documents and harmonization initiatives have led to improved detection of LA.
• Eliminating future diagnostic errors requires optimizing expert guidance and clinician education.

## 1 Introduction

Antiphospholipid syndrome (APS) represents a serious clinical condition in which patients may be at high risk for thrombosis, pregnancy/fetal morbidity/mortality, or other multisystem clinical presentations [1]. The latest APS classification criteria [2] identify patients as having definite APS based on a clinical and laboratory scoring system as the entry criterion and persistent presence of certain antiphospholipid antibodies (aPL). These aPL tests are also more broadly used to diagnose or exclude APS [1,3,4]. These aPL tests include the lupus anticoagulant (LA), anticardiolipin antibodies (aCL), and anti–beta2-glycoprotein I antibodies (aβ2GPI). The aCL and aβ2GPI tests are immunological assays, and the presence of either immunoglobulin (Ig)G or IgM for aCL and/or aβ2GPI, confirmed after at least 12 weeks, can help diagnose APS. These tests are often performed in immunology laboratories using serum or plasma samples. These immunological assays are “calibrated” against a standard, and the titer of aPL can be identified [[3], [4], [5], [6], [7], [8]]. Higher titer antibodies are more often associated with adverse clinical events, such as thrombosis, than lower titer antibodies [3].

In contrast, LA is identified using clot-based assays, using plasma (not serum), and generally within hematology laboratories. Because LA is identified using clot-based assays, it is subject to considerable preanalytical and analytical issues that challenge accurate detection or exclusion of LA. In particular, several anticoagulants, given as therapy to treat and prevent the pathophysiological consequences of aPL, especially thrombosis, interfere with LA assays and can generate false-positive or false-negative LA findings [9]. Additional preanalytical issues include sample collection and transport, with possibility of compromising LA detection due to underfilled or clotted collection tubes. Analytical issues include the different tests employed by laboratories, as well as the approaches used [10].

To overcome the possibility of diagnostic errors associated with LA testing, several strategies can be employed. For anticoagulant interference, various possibilities exist, including use of anticoagulant neutralizers, switching patients to less-interfering anticoagulants, or collecting patients after holding anticoagulants for an appropriate time period based on drug and renal clearance. Various expert or consensus guidelines are available, which provide helpful guidance on LA testing [4,9,11,12]. In this narrative review, we take a look at the good, the bad, and the ugly of LA testing, primarily focusing on the last 10 years.

## 2 Tests used to Diagnose/Exclude LA or Otherwise Help with Differential Analysis—A Complicated Landscape

**Figure 1:**
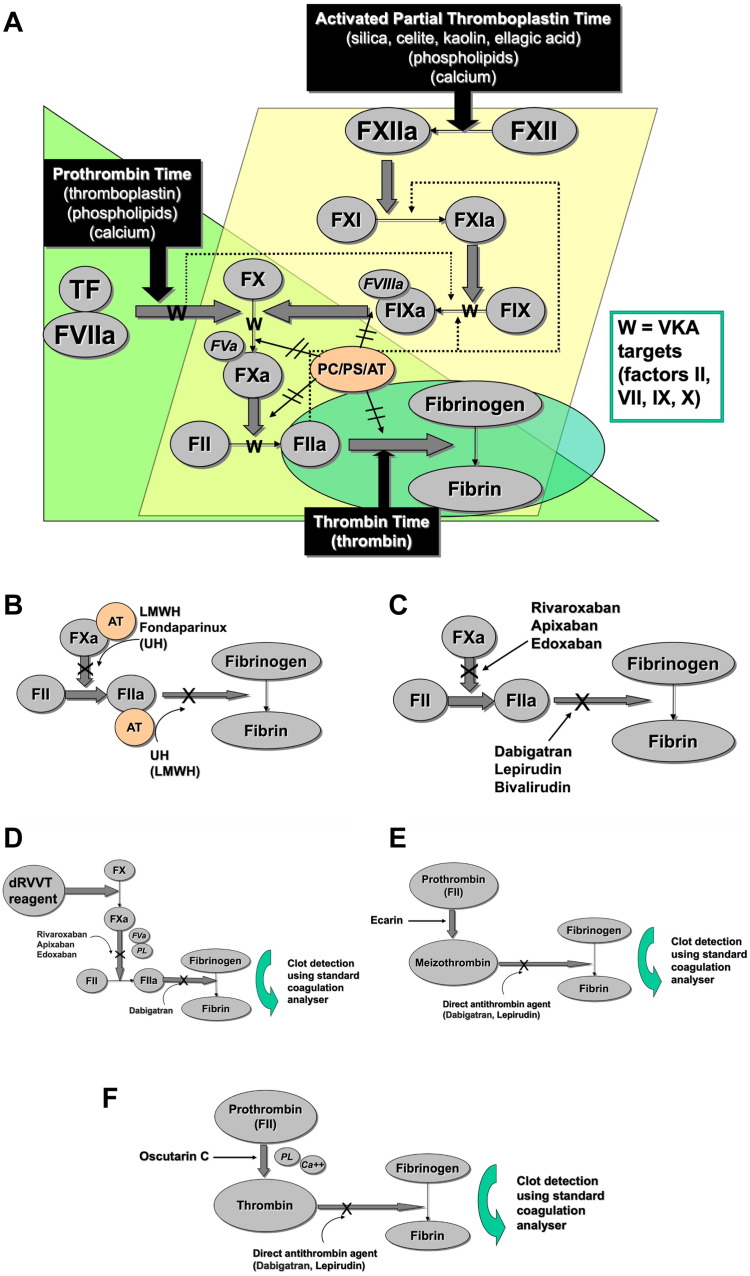
A pictorial representation of the main laboratory tests used in investigation of lupus anticoagulants or in differential investigations (eg, to assess presence of various anticoagulants). The figure also summarizes inhibition points for various anticoagulants. (A) A pictorial representation of the prothrombin time, activated partial thromboplastin time, and thrombin time, identifying regions of these pathways affected by vitamin K antagonists (VKA) such as warfarin (“W”), which inhibits production of fully functional factors (F)II, VII, IX, and X. Inhibition sites for antithrombin (AT), protein C (PC), and protein S (PS) are shown for comparison. (B) A pictorial representation of the terminal stages of the common pathway of coagulation (eg, from panel A) identifying the regions inhibited by low-molecular-weight heparin (LMWH), unfractionated heparin (UH), and fondaparinux. LMWH mainly inhibits FXa, and UH mainly inhibits FIIa (ie, thrombin). These effects are indirect and require AT as a cofactor. (C) A pictorial representation of the terminal stages of the common pathway of coagulation (eg, from panel A) identifying the regions inhibited by direct inhibitors of FXa (eg, the direct oral anticoagulants [DOACs] rivaroxaban, apixaban, and edoxaban) and FIIa (eg, the DOAC dabigatran and the parenteral agents, lepirudin, and bivalirudin). (D) A pictorial representation of the dilute Russell viper venom time (dRVVT) identifying the potential regions inhibited by the DOACs. Although dRVVT assays are potentially also affected by UFH and LMWH, most manufacturers now use heparin neutralizers capable of neutralizing therapeutic levels of heparin. The dRVVT is also affected by VKAs (ie, at FII and FX). (E) A pictorial representation of the ECT. This assay employs direct activation of FII and is thus not affected by the anti-Xa agents (eg, rivaroxaban and apixaban) but is affected by anti-IIa agents (eg, dabigatran). (F) A pictorial representation of the Taipan snake venom time. Like the ECT, this assay also employs direct activation of FII (via the venom component Oscutarin C) and is thus not affected by the anti-Xa agents (eg, rivaroxaban, apixaban, and edoxaban) but is affected by anti-IIa agents (eg, dabigatran). The venom can also activate undercarboxylated prothrombin resulting from VKA therapy and so can “overcome” the interference from VKA therapy. PL, phospholipid.

Before evaluating the good, bad, and ugly of LA testing, it is helpful to identify the tests that may be performed by laboratories in such settings. Current LA testing guidelines recommend use of at least 2 tests based on different assay principles before excluding LA, in particular recommending the activated partial thromboplastin time (aPTT) and dilute Russell viper venom time (dRVVT) [11,[13], [14], [15]]. The aPTT is based on contact activation, and the dRVVT is based on factor (F)X activation (Figure 1) [16,17]. Additional tests are used in some situations, for example, when there is a strong suspicion of LA but aPTT and dRVVT are inconclusive or if there is assay interference (eg, from clinical anticoagulants). Such tests include the Taipan snake venom time (TSVT), the Ecarin clotting time (ECT), and the dilute prothrombin time (PT) [[18], [19], [20]]. Instead of the aPTT, an alternative sensitive assay based on a similar principle (ie, contact activation) is the silica clotting time [16]. Additional tests used for LA investigation that have been particularly favored in the past are the kaolin clotting time (also based on contact activation) and LA confirmation using the platelet neutralization procedure. Although not used to identify LA per se, the standard (“undiluted”) PT is also often used, primarily to help identify the possibility of anticoagulant presence [12]. The aPTT may also be involved in assessing for anticoagulant presence, as well as the thrombin time [21]. It is also possible that laboratories use different aPTT reagents to assess for LA vs to assess for anticoagulant presence.

Furthermore, when used for LA testing, the recommendation is for the dRVVT and aPTT to be performed as duplicate tests using a screening test sensitive to the presence of LA (ie, aPTT and dRVVT screen, typically having a low concentration of phospholipid), and if these are prolonged, the tests are then repeated using a confirmatory test relatively insensitive to the presence of LA (ie, aPTT and dRVVT “confirm,” typically having a high concentration of phospholipid) [11,13,16,17].

But there is even more complexity: since LA represents an inhibitory (“antibody-mediated”) effect, the laboratory also employs mixing studies to differentiate such an inhibition (where samples yielding prolonged screening test times still yield prolonged test times even upon mixing with normal plasma) vs a clotting factor deficiency (where samples yielding prolonged screening test times then yield normal test times upon mixing with normal plasma) [11,13,16,17,22]. Such mixing studies are also used by the laboratory outside the setting of LA (eg, to help determine factor deficiency vs anticoagulant effects on coagulation tests) [22]. How mixing studies are used in LA detection/exclusion may differ from laboratory to laboratory and also according to LA guidelines [11,13,23].

In summary, there is a large number of potential tests used by the hemostasis laboratory, both to evaluate for LA as well as to provide differential explanations for prolonged clotting times (including anticoagulants, factor deficiencies, and factor inhibitors). For LA investigation, laboratories may use different test “panels” and also different test approaches (eg, when mixing tests are performed). Laboratories also use reagents from many different manufacturers/suppliers, different reference ranges, and different cutoff values and approaches to determine LA positivity vs negativity, and therefore, often yield different testing landscapes in different laboratories [10]. There is also a large number of clinical anticoagulants in use, and these may affect different assays in different ways (Table 1, Figure 1) [9,12].

**Table 1:** Preanalytical, analytical, and postanalytical issues in testing for lupus anticoagulant.

Issue	What tests are affected?	Suggested solution…	… With caveats
**Preanalytical issues**			
**Anticoagulant therapy**	Depends on anticoagulant and level	a. Test LA whilst at anticoagulant trough b. Cease anticoagulant therapy for short duration to test LA. c. Change anticoagulant therapy to one that is less interfering in LA testing, for example, LMWH. d. Incorporation of information on the patient’s anticoagulation at the time of clinical test request is mandatory [11]	a. Even low concentrations of some anticoagulants, especially DOACs, will affect some clot-based assays, so false LA events remain possible. b. Some clinicians may be hesitant to do so, perhaps fearing the potential of an (unlikely) adverse clinical event c. Some clinicians and patients may be hesitant to change anticoagulants. d. Not all clinicians will comply, and some will simply not know
UFH	aPTT, dRVVT, (TT; PT if exceeds neutralizer ability)	a. For dRVVT, can use reagents with heparin neutralizers. b. For aPTT, can use CaCl_2_ reagent with heparin neutralizers [24]	a. Possibility that high heparin level may exceed neutralizer ability. b. Reagents may not be available in certain localities
LMWH	(aPTT, dRVVT)		
VKA (eg, warfarin)	aPTT, dRVVT, (PT)	a. Mixing 1:1 with normal plasma can dilute out the VKA effect. b. Consider use of TSVT with ECT as a confirmatory test	a. Latest LA guidance does not recommend “mixing” as a simple strategy since “false-positive or -negative” LA is possible [12]. b. Test/reagents not widely available and not available in all geographic localities
DOACs	aPTT, dRVVT, (PT)	a. As per general solutions for anticoagulants b. Use DOAC neutralizers c. Consider use of TSVT with ECT as a confirmatory test for anti-Xa DOACs	a. As per general caveats for anticoagulants b. Extra time and sample requirements; neutralizers may not neutralize all DOAC interference; concern that neutralizers may have unknown effects on clot tests in some patients. Need additional plasma to enable all testing. c. Test/reagents not widely available and not available in all geographic localities; will not address anti-IIa DOACs
**Sample collection (timing)**	Potentially, all LA tests	Timing of LA testing in regard to acute thrombotic episodes or pregnancy should be considered. LA testing should be generally avoided in the acute phase or during pregnancy (due to elevation in acute phase proteins or coagulation proteins potentially affecting LA assays) [11].	Testing at these times may be unavoidable, and if so, should be taken into account when interpreting test results, also considering repeat testing for confirmation after the acute phase or pregnancy. Testing during the acute phase may be required (in patients with suspected catastrophic APS) or desirable (in new stroke patients suspected of having APS). If LA testing is performed in these situations or in pregnancy, repeat testing should be undertaken for confirmation after the acute phase or after 6 weeks postpartum.
**Sample collection (underfilled samples, clotted samples)**	aPTT, dRVVT, (PT)	Education—ensure collection staff know the requirements	Difficult to educate all staff collecting blood (especially clinical staff outside the scope of local pathology training)
**Sample processing (poor transport, poor centrifugation, poor freezing)**	aPTT, dRVVT, (PT)	Education—ensure staff know the requirements	Difficult to educate all staff collecting blood (especially staff outside the facility—referred samples)
**Analytical issues** (type and panel of tests performed, sequence of testing, instruments)	aPTT, dRVVT, (PT)	Education—follow guidelines	Some tests/reagents may not be available in some locations
**Post analytical** (cutoff values, reference ranges, interpretation, reporting)	aPTT, dRVVT, (PT)	a. Education—follow guidelines b. LA results should always be related to the results of aCL and aβ2GPI to assess the risk profile, and overall results should be interpreted in a clinical context and knowledge of ongoing treatment [11].	

aPTT and dRVVT may be employed to assess LA; other tests may be employed to assess presence of anticoagulant or to assess for potentially compromised LA sample (eg, clotted sample).

aβ2GPI, anti-beta2-glycoprotein I antibodies; aCL, anticardiolipin antibodies; APS, antiphospholipid syndrome; APTT, activated partial thromboplastin time; DOAC, direct oral anticoagulant; dRVVT, dilute Russell viper venom time; ECT, Ecarin clotting time; LA, lupus anticoagulant; LMWH, low-molecular-weight heparin; PT, prothrombin time; TSVT; Taipan snake venom time; TT, thrombin time; UFH, unfractionated heparin; VKA, vitamin K antagonist.

## 3 The Ugly of LA Testing—Preanalytical Issues

**Figure 2:**
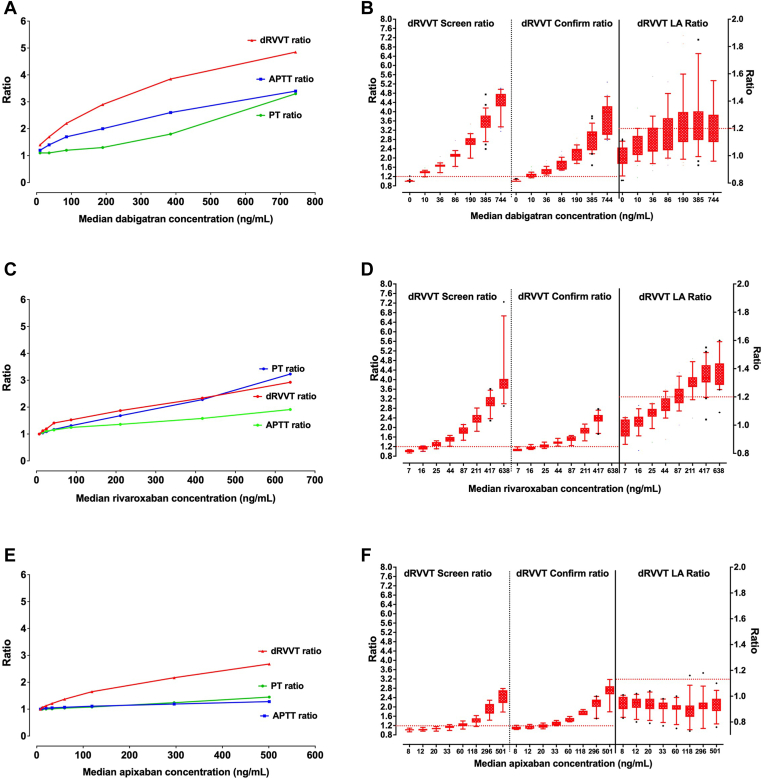
The effect of direct oral anticoagulants (DOACs) on the main tests used in lupus anticoagulant (LA) testing or in assessing for potential anticoagulant effects. (A) Differential effects of dabigatran on prothrombin time (PT), activated partial thromboplastin time (aPTT), and dilute Russell Viper venom time (dRVVT) screen. Data are shown as median test results from participants of the Royal College of Pathologists of Australasia Quality Assurance Program (RCPAQAP) from a survey of test practice, as published in 2015 [22]. (B) Differential effects of dabigatran on dRVVT screen and confirm, as well as screen/confirm ratio. Data are shown as box and whiskers showing 10th to 90th percentiles of results from participants of the RCPAQAP from a survey of test practice, as published in 2015 [22]. Note the wide scatter of results for screen, identifying different dRVVT screen reagents have differing sensitivity to dabigatran. The end result, in general, is an increase in screen/confirm ratio, and thus, potential for false positive LA. (C) Differential effects of rivaroxaban on PT, APTT, and dRVVT screen. Data are shown as median test results from participants of the RCPAQAP from a survey of test practice, as published in 2016 [23]. (D) Differential effects of rivaroxaban on dRVVT screen and confirm, as well as screen/confirm ratio. Data are shown as box and whiskers showing 10th to 90th percentiles of results from participants of the RCPAQAP from a survey of test practice, as published in 2016 [23]. Note the wide scatter of results for the screen, identifying different dRVVT screen reagents having differing sensitivity to rivaroxaban. The end result, in general, is an increase in screen/confirm ratio, and thus, potential for false positive LA. (E) Differential effects of apixaban on PT, APTT, and dRVVT screen. Data are shown as median test results from participants of the RCPAQAP from a survey of test practice, as published in 2016 [23]. (F) Differential effects of apixaban on dRVVT screen and confirm, as well as screen/confirm ratio. Data are shown as box and whiskers showing 10th to 90th percentiles of results from participants of the RCPAQAP from a survey of test practice, as published in 2016 [23]. Despite differential assay variability in sensitivity to apixaban, the end result, in general, is a decrease in screen/confirm ratio, and thus potential for false negative LA in an LA-positive patient. For B, D, and E, the red horizontal dashed line at a ratio of 1.2 represents the “default” cutoff for positive/negative LA findings, as used by the majority of RCPAQAP participants [10,[26], [27], [28]]. The data reflects normal pool plasma spiked with various concentrations of the DOACs; as the DOAC concentration increases, a higher proportion of dRVVT screen and confirm test times exceed the cutoffs. For dabigatran and rivaroxaban, the effect is greater on the dRVVT screen, and so the screen/confirm ratio also goes up with increasing DOAC level, being suggestive of LA (ie, ratio > 1.2) at “within-therapy” DOAC ranges. Instead, for apixaban, the effect was greater on the dRVVT confirm, and so the screen/confirm ratio tended to go down with increasing DOAC level.

One of the main problems with LA testing is the requirement for sodium citrate anticoagulated plasma samples, preferably collected and transported in ideal settings, from a patient not taking any medication that may interfere with the clot-based tests, especially anticoagulants. Unfortunately, the risk of testing patients for LA whilst they are on anticoagulant therapy is very high [25]. First, patients are investigated for LA usually because they have had a clinical event such as a thrombosis (“symptomatic” APS) or else they have yielded an abnormal clotting assay during a prior coagulation screening investigation (potentially “APS carriers” or “asymptomatic APS”). In these situations, anticoagulants could be either a cause of a false LA or the cause of the initial prolonged clotting assay (eg, aPTT) leading to subsequent investigation of a LA. The presence of anticoagulants compromises the performance of many clot-based assays, including LA tests. All anticoagulants, including low-molecular-weight heparin (LMWH), unfractionated heparin (UFH), vitamin K antagonists (VKA) such as warfarin, and the newer direct oral anticoagulants (DOACs), can interfere with clot-based assays (Table 1, Figure 1) [9,12,21]. Even if a patient is not on an anticoagulant during the initial test analysis, the patient needs to be retested after at least 12 weeks to assess LA persistence, at which time they are likely to be on anticoagulant therapy. Unfortunately, clinicians requesting LA tests often do not understand that anticoagulants have substantial adverse effect on laboratory tests, and they not only order LA tests on patients on anticoagulant therapy but often do not convey this important clinical information to the laboratory performing the tests. This situation risks both false-positive and false-negative LA test findings [9,11,21,25]. All DOACs can lead to false-positive LA test patterns (rivaroxaban, apixaban, edoxaban, and dabigatran), and some like apixaban can also cause false-negative LA test patterns (Figure 2) [9,12,21,[25], [26], [27],^29^].

Additional preanalytical issues include sample collection and processing (Table 1) [30,31]. Breakdowns in these processes can lead to compromised samples for testing. The correct fill for a sodium citrate blood collection, as required for LA tests, is 9 parts blood to 1 part citrate. Underfilled coagulation tubes, due perhaps to a difficult collection, lead to an excess of sodium citrate anticoagulant, which both overdilutes the plasma and also leads to excess citrate that may compromise the recalcification required to promote clotting in the LA tests. Poor collection and delayed blood tube mixing may also lead to clotting within the blood collection tube, which will then compromise subsequent LA tests, usually due to prolonged or nonclottable samples. LA samples also need to be processed correctly, which includes appropriate transportation from the collection site to the test site and then appropriate centrifugation to prepare double-spun plasma [11]. Finally, since LA testing is often performed in specialized laboratories in batches to maximize efficiency, samples are often frozen and then thawed prior to testing. The type of freezers used, the duration of freezing, and the process of thawing may all impact LA test results.

## 4 The Bad of LA Testing—Analytical Issues

As mentioned in the section “Tests used to diagnose/exclude LA or otherwise help with differential analysis—a complicated landscape,” a large battery of tests is available in hemostasis laboratories to diagnose/exclude LA or to otherwise assess for potential presence of anticoagulants, factor deficiencies, or inhibitors. These tests originate from a large base of potential manufacturers/suppliers, use different reagent formulations, are differentially affected by LA/anticoagulants/inhibitors/factor deficiencies, and are performed on a variety of instruments, again from a large base of potential manufacturers/suppliers (Table 2) [10]. These tests have a variety of reference ranges; even the same tests (eg, aPTT or dRVVT) from the same manufacturer may have different reference ranges in different laboratories based on the use of different instruments or different tested populations. Similarly, different laboratories may employ different processes for determining assay cutoffs for LA positivity, including assay ratios (eg, screen/confirm) or percent correction. For assay ratios, most laboratories, but not all, use “normalized” ratios, which are adjusted for the assay variation between screen and confirm assays. Even if essentially using the same approach (eg, normalized ratios), different laboratories may have established these in different ways, using different approaches, different normal and LA test samples, and so on, and so generating different assay ratios. It also seems that some laboratories are confused regarding the characterization of their reagents as a screen or confirm reagent [10]. Thus, although most participants of one local external quality assessment (EQA) program list HemosIL Synthasil and Siemens Actin FSL as aPTT-screening reagents for LA, these same reagents are also occasionally (incorrectly) listed as aPTT-confirm reagents for LA [10].

**Table 2:** Analytical variables in lupus anticoagulant testing. Summary of recent data are from the Royal College of Pathologists of Australasia Quality Assurance Program.^a^

Tests used by participants	From these manufacturers (*n* = participant numbers)
dRVVT screen	Haematex dRVT LS (*n* = 1)
	IL LAC screen (*n* = 43)
	Siemens LA 1 screen (*n* = 43)
	Stago Staclot DRVVT (*n* = 36)
dRVVT confirm	Haematex dRVT LR (*n* = 1)
	IL LAC confirm (*n* = 41)
	Siemens LA 2 confirm (*n* = 35)
	Stago Staclot DRVVT (*n* = 36)
aPTT screen	HemosIL aPTT SP (*n* = 1)
	HemosIL Synthasil (*n* = 26)
	Intrinsin-LS/Cephen 2.5 LS (*n* = 20)
	Siemens Actin FSL (*n* = 44)
	Siemens Pathromtin SL (*n* = 2)
	Stago PTT Automate (*n* = 1)
	Stago PTT-LA (*n* = 20)
	Stago Tcoag TriniCLOT aPTT HS (*n* = 6)
	Stago Tcoag TriniCLOT aPTT S (*n* = 4)
	Tcoag TriniCLOT Automated aPTT (*n* = 1)
aPTT confirm^b^	Intrinsin-LR/Cephen 5 (*n* = 15)
	Siemens Actin FS (*n* = 18)
	Siemens Actin FSL (*n* = 3)
	HemosIL Synthasil (*n* = 3)
	Stago Tcoag TriniCLOT aPTT HS (*n* = 1)
Stago Staclot-LA^c^	*N* = 10
SCT screen and confirm	IL (*n* = 21)
KCT	Haematex SACT (*n* = 2)
	R2 Diagnostics LupoTek KCT (*n* = 2)
	In-house reagent (*n* = 2)

aPTT, activated partial thromboplastin time; dRVVT, dilute Russell viper venom time; KCT, kaolin clotting time; SCT, silica clotting time.

a Table shows number of participants using particular tests or reagents. Almost all participants use dRVVT- and aPTT-based screening but from a variety of manufacturers (especially for aPTT). Only a third of participants use an aPTT-confirm reagent. A small proportion of participants use alternate assays, such as SCT and KCT. The use of other assays, such as Taipan snake venom time and Ecarin clotting time, is not captured by the Royal College of Pathologists of Australasia Quality Assurance Program but is anticipated to be very few laboratories. Data are mostly summarized from end of 2022 (sample 6) but may include earlier data for showing extent of variability; sometimes, participants omit to include information about the reagents they use, so numbers in screen vs confirm reagents do not always tally.

b Sometimes, laboratories identify the same reagents as both LA screening and confirm reagents (eg, Siemens Actin FSL and HemosIL Synthasil).

c aPTT-based hexagonal phospholipid screening and confirmatory assay for LA.

To summarize here, given the large number of analytical variables employed in the methods used to identify or exclude LA, different laboratories testing the same sample for LA may come to a different conclusion regarding the presence or absence of LA (ie, positive vs negative for LA), especially if the sample contains “weak” LA, thereby yielding test results close to cutoff values, or else may falsely identify LA in an anticoagulated sample (especially possible with DOACs) [10,32]. Finally, different laboratories may report LA findings in a variety of ways. This lack of assay and method standardization or harmonization complicates the LA testing landscape.

## 5 The Good of LA Testing

The reader should not at this stage conclude that LA testing is all doom and gloom. There are some positive messages to take home.

### 5.1 Expert guidance

First and foremost, the International Society on Thrombosis and Haemostasis (ISTH) Scientific Standardization Committee (SSC) on LA is very active and provides excellent advice around LA testing. Indeed, the ISTH SSC on LA has published many guidance documents in recent years [11,12,20,[33], [34], [35]], building on initiatives and guidance from past ISTH SSCs [23,36,37]. In terms of LA testing, the latest 2 main guidance documents provide an update for LA detection and interpretation [11] and for LA detection in anticoagulated patients [12] (Table 3). There are also LA guidance documents from other expert groups [[13], [14], [15]], although their earlier date of release may limit their current usefulness subsequent to the emergence of the DOACs. Expert guidance from the ISTH SSC also extends to considering alternatives to aPTT and dRVVT in patients on DOACs, such as the TSVT with ECT as the confirmatory test in patients on VKAs or anti-Xa agents [20].

**Table 3:** Summary of points covered in the latest ISTH SSC guidance documents.

Update of the guidelines for lupus anticoagulant detection and interpretation in Devreese et al. [11]. • Latest in series of ISTH SSC guidelines for LA detection/interpretation. • Patient selection for LA testing has been expanded. • More detailed recommendations on how to handle testing in anticoagulated patients and timing of testing. • Routine coagulation tests are advised for information on patient anticoagulation background (when necessary, anti-Xa activity measurement for heparins or specific assays for DOACs). • Three-step procedure; dRVVT and aPTT essentially unchanged. • Silica remains the preferred activator for aPTT, but ellagic acid is not excluded. • Recommend simultaneous performance of mixing and confirmatory steps for each sample with prolonged screening test. • Confirmatory steps can also be performed on a mixture of patient plasma and normal pooled plasma. • Cutoff values should be established in-house on at least 120 normal or transference of manufacturer’s cutoffs as an alternative. • Reporting of results has not been changed, although more attention is paid to what clinicians should know. LA detection in anticoagulated patients in Tripodi et al. [12]. • Anticoagulants interfere with LA tests (= occasional false-positive or false-negative LA). • Some commercial tests include heparin neutralizers (UFH or LMWH up to 1.0 U/mL; dRVVT). • LA tests are less affected by LMWH, but caution is still needed for result interpretation. • VKAs may affect LA detection, but dilution of test plasma into pooled normal plasma is “not a reliable solution as false-negative or false-positive LA may occur.”^a^ • DOACs affect LA detection; thus, it is not recommended to attempt LA detection in these patients.^b^ • DOAC adsorbents are a promising solution and should be further investigated. • TSVT/ECT tests may be a solution for VKAs and anti-FXa DOACs, but independent evidence on their value and standardized kits is needed.

aPTT, activated partial thromboplastin time; DOAC, direct oral anticoagulant; dRVVT, dilute Russell viper venom time; ECT, Ecarin clotting time; ISTH SSC, International Society on Thrombosis and Haemostasis Scientific Standardization Committee; LA, lupus anticoagulant; LMWH, low-molecular-weight heparin; TSVT; Taipan snake venom time; UFH, unfractionated heparin; VKA, vitamin K antagonist.

a However, as stated in the Devreese et al. [11] guideline, “if the INR is between 1.5 and <3.0, a 1:1 dilution of patient plasma and PNP can be considered” (Table 2 of the guideline).

b However, as stated in the Devreese et al. [11] guideline, “if feasible to temporarily interrupt DOAC anticoagulation (on a pragmatic, empirical basis at least 48 hours after the last dose, and longer in patients with renal impairment), LA testing can be performed, with the DOAC level checked alongside the LA test.”

Also important to note here is that although DOACs affect LA tests, many patients with APS, and thus likely to be assessed for LA, may be transitioned away from DOACs and onto VKA and other therapies, according to preferred management strategies (eg, those at highest risk of thrombosis or triple positive for aPL) [35].

### 5.2 Anticoagulant neutralizers

As noted previously, all anticoagulants can affect clot-based assays. Indeed, we use this anticoagulant sensitivity to effectively monitor and manage some patients on anticoagulants (eg, the PT converted to the international normalized ratio [INR] for monitoring VKA therapy and the aPTT to monitor UFH therapy) [38,39]. However, the presence of VKAs and UFH will affect most assays, including the PT, aPTT, and dRVVT. Since we use the PT (as the INR) for monitoring VKA, the aPTT to monitor UFH, and the dRVVT for assessing LA, manufacturers may add heparin neutralizers to PT and dRVVT (but not aPTT) reagents to make them insensitive to heparin, and thus, this mostly avoids the possibility of heparin interference in LA testing using the dRVVT but does not address the possibility of heparin interference in LA testing using the aPTT. Nevertheless, laboratories should not be complacent, as reagents with neutralizers will only neutralize heparin within therapeutic levels, and thus, supratherapeutic levels may still interfere with LA testing by dRVVT.

There are no easy means of “neutralizing” VKA effects within aPTT and dRVVT assays, so in the past, laboratories resorted to use of mixing studies to overcome the factor deficiency state induced by VKAs. That is, patient samples for aPTT and dRVVT, prolonged because of a VKA effect, should become normal after mixing 1:1 with normal plasma [[21], [22], [23]]. Thus, any prolongation remaining after mixing would be due to an inhibitor such as LA (although an anticoagulant effect [eg, DOAC] or a factor inhibitor may need to also be excluded). Although mixing studies for normalizing VKA effects was encouraged in earlier guidelines [13,23,36,37], they were not unanimously supported in the latest guidelines [11,12] since there was a perceived risk of false-positive and false-negative LA findings using this approach. Nevertheless, the guidance does caveat this risk and states that in VKA-treated patients, “if the INR is between 1.5 and <3.0, a 1:1 dilution of patient plasma and pooled normal plasma (PNP) can be considered” (Table 2 of the guideline) [11]. We also confirm that in our own laboratories, we still use this strategy to help overcome VKA effects as a lesser evil to alternatives. However, the greatest risk currently is the influence of DOACs on LA tests [9,11,12,21,23,[25], [26], [27],[29], [30], [31]].

**Figure 3:**
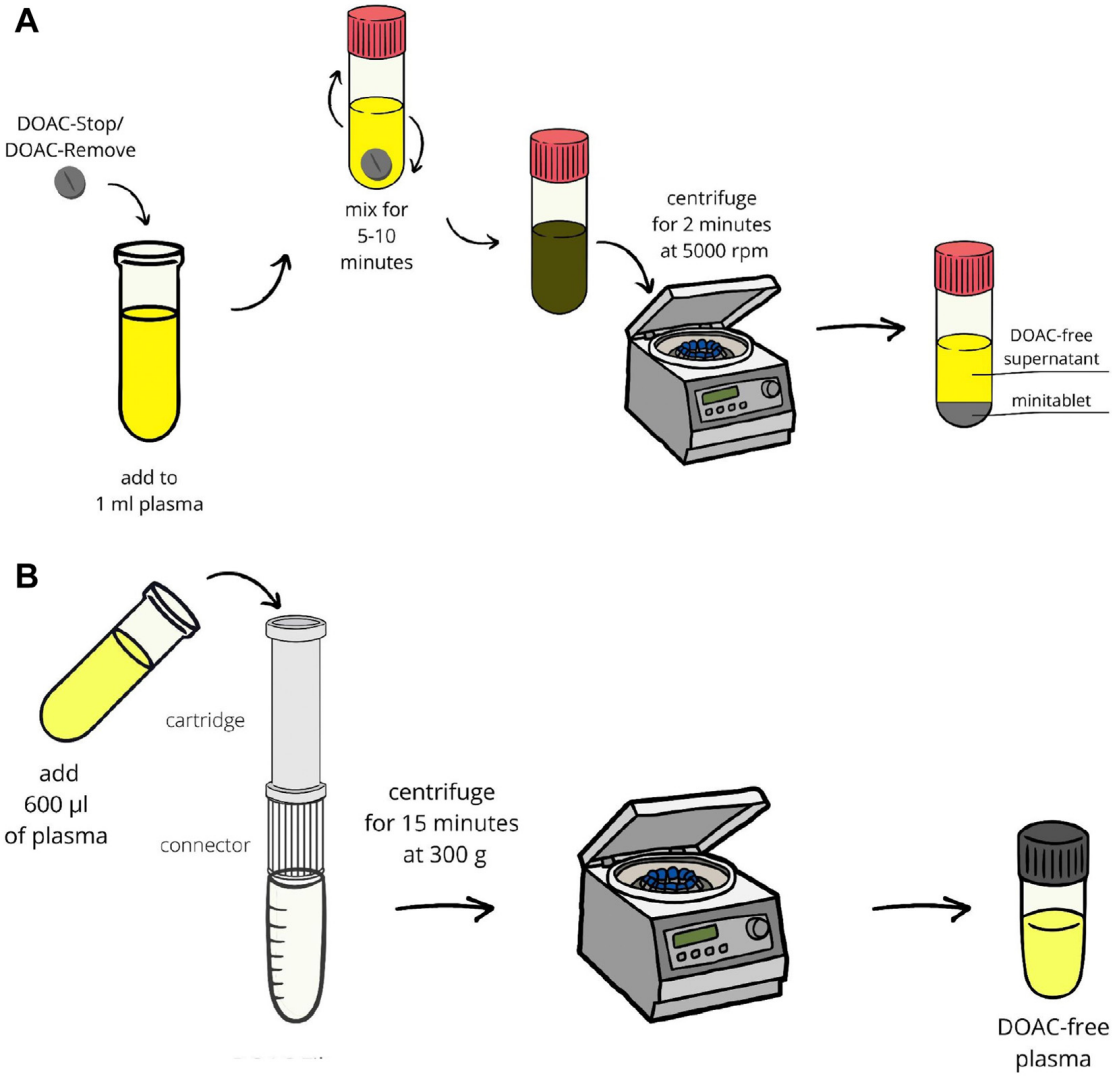
Methods for neutralization of direct oral anticoagulant (DOAC) interference in coagulation assays. *Source:* Figures reproduced from [40]. Permission for reuse according to Creative Commons Attribution 4.0 International License (http://creativecommons.org/licenses/by/4.0/).

All DOACs have an effect on clot-based assays, but the effect depends on the anticoagulant, its level, and the assay used. As an educational example, Figure 2 shows a general trend for the 3 main DOACs (rivaroxaban, apixaban, and dabigatran) in use, using data from Australasia [26,27]. For dabigatran, the dRVVT is affected more than the aPTT, which is affected more than the PT (Figure 2A). For rivaroxaban, the PT and dRVVT are affected similarly and more than the aPTT (Figure 2C). For apixaban, the dRVVT is affected, but the PT and aPTT are hardly so (Figure 2E). Nevertheless, all these DOACs show some interference in the dRVVT assay, and potentially also in the aPTT assay, and so can affect LA testing. Moreover, for dabigatran and rivaroxaban, the dRVVT screen is affected more than the dRVVT confirm (Figures 2B, D), and so there is a potential for increased dRVVT screen/confirm ratio with increasing DOAC concentration, such that ratios can exceed the cutoff for positivity and give rise to false-positive LA at within-therapy DOAC ranges. The pattern with apixaban is different, and for the Australasian data, the dRVVT confirm was affected more than the dRVVT screen, and so there is a potential for a decreased dRVVT screen/confirm ratio with increasing DOAC concentration, such that reduced ratios can give rise to false-negative LA in an LA-positive patient at within-therapy DOAC ranges (Figure 2F). However, others have reported the possibility of false-positive LA with apixaban [29]. Unfortunately, no manufacturer is currently able to provide a within-reagent neutralizer solution for DOACs, although DOAC neutralizers are available from a number of manufacturers/suppliers [9]. These are primarily comprised of activated charcoal reagents that are mixed with the plasma sample and then need to be removed, typically by centrifugation, to prove a plasma sample is “depleted” of DOAC (Figure 3). Since it is unclear whether these agents may have unknown effects on some plasma samples, it is recommended to only use these agents on samples with high suspicion of DOAC presence (eg, as conveyed by the requesting clinician or as suggested by patterns of coagulation test results [21]). Numerous studies have shown these to be effective against DOACs, removing all to most of the DOACs present and leaving a sample that is then essentially “DOAC-free” for LA testing (reviewed in [9]). However, these processes may add additional handling time for each sample so treated, and so the process may not be feasible in some very busy laboratories. As an example, our laboratories process batches of ∼40 samples several times a week for LA testing, and processing all samples with a DOAC neutralizer could require an additional 13 hours of technologist time per week. Even targeting specific samples may compromise our ability to perform our standard LA batches in a single day. There also remain concerns regarding sufficient sample quantity since at least 1 mL of plasma is required to perform all LA testing, and thus, larger volumes are required if DOAC neutralization is also required. Also, some have raised concerns that in some patients with high DOAC concentrations, DOACs may not be fully neutralized, thus leaving some potential for residual DOAC interference in some cases.

The Westmead experience with these DOAC neutralizers is with the original reagent DOAC-Stop (Haematex, Sydney), manufactured in Australia, which we showed was effective to fully neutralize rivaroxaban in an EQA setting [32]. DOAC-Stop neutralization of rivaroxaban was even more effective than that of andexanet alfa [32]. In brief, a plasma pool was spiked with a within-therapy concentration of 200 ng/mL rivaroxaban, and then this material was subjected to DOAC-Stop or andexanet alfa. Samples were sent to Royal College of Pathologists of Australasia Quality Assurance Program (RCPAQAP) participants enrolled in the LA test module. All participants (ie, 100%) identified the pooled plasma as LA negative, and most (ie, >80%) identified the rivaroxaban spiked sample as either LA positive or as consistent with the presence of a test-interfering substance. The sample treated with DOAC-Stop was identified by all participants (ie, 100%) as LA negative, confirming the total neutralization of the rivaroxaban-induced interference/false-positive LA, whereas the sample treated with andexanet alfa was identified to contain a test-interfering substance by nearly 40% of participants (due to incomplete neutralization of the rivaroxaban effect, plus a potential interference by andexanet alfa).

More recently, the manufacturer of DOAC-Stop has generated an enhancement to the process of DOAC neutralization with a product they have called DOAC-Stop Liquid. This product requires less time for dispersal into plasma and thus more quickly neutralizes DOACS (within 2 minutes in most cases, compared with 5 to 10 minutes for the standard product) [28]. This product was able to neutralize up to 2000 ng/mL of the assessed DOACS (apixaban, rivaroxaban, and dabigatran) in the assessed hemostasis assays [28]. Moreover, there was no need to centrifuge the material from the solution in the evaluated mechanical clot detection device [28]. Although further evaluation and confirmation are needed, this may reduce the process from the current 10 to 15 minutes per sample to ∼5 minutes per sample for mechanical clot detection systems [28].

In any case, DOAC neutralization should in general be accompanied with other strategies. Should testing in absence of anticoagulant not be feasible, then testing at trough period of treatment with heparin (UFH or LMWH) or DOAC would be preferable over testing at peak or “steady state.” Alternatively, if feasible, high-risk patients on DOACs could be switched to LMWH, which represents a lower risk for LA interference. In such cases, testing for anticoagulant activity should be performed to inform interpretation of the LA. However, our preference remains that testing should ideally be done in the absence of anticoagulant therapy, especially DOAC therapy since even trough levels can affect LA assays [[41], [42], [43]]. Moreover, performance of additional anticoagulant activity assays adds time, cost, and complexity to the process and may be difficult in small or busy laboratories; moreover, this additional testing may not be reimbursed to the laboratory since tests performed were not specifically requested by the clinician. Finally, laboratories can only advise requesting clinicians, who may not follow such advice.

### 5.3 Assay harmonization initiatives

There are several initiatives aiming to standardize and harmonize LA testing and reporting. First, ISTH SSC guidance documents [11,12,20,23] and also expert/consensus guidance from other groups [[13], [14], [15]] have been an impetus toward harmonization of LA testing and reporting, essentially providing guidance on which tests and test approaches to use, and the majority of laboratories seem to be following such guidance. Other initiatives include EQA/proficiency testing from a variety of organizations. These essentially comprise surveys of practice and proficiency assessment of homogeneous samples across a large number of laboratories, which then provides a scatter of results that then can be interpreted into what tests/approaches work well and what do not. As an example, the latest report from the RCPAQAP has recently been published [10], as have prior reports from the RCPAQAP and other EQA providers/surveys of practice [[44], [45], [46], [47], [48], [49], [50], [51], [52]].

Also worth noting are initiatives that aim to harmonize test processing across large laboratory networks [[53], [54], [55], [56]]. Finally, we would like to highlight several initiatives that aim to harmonize diagnostic criteria for inclusion of patients into clinical trials, as well as the increasing number of data reports from APS ACTION (AntiPhospholipid Syndrome Alliance for Clinical Trials and InternatiOnal Networking) group utilizing “homogeneous” diagnostic criteria [[57], [58], [59], [60], [61], [62], [63], [64], [65], [66], [67]]. The recent publication of the latest classification criteria for APS [2] will hopefully drive further international effort to harmonize research trial enrolment and outcomes.

### 5.4 ISTH Congress Report

Several relevant presentations were delivered at the latest ISTH 2023 Congress held in Montreal, Canada, in July of 2023, as summarized in Table 4 [24,[68], [69], [70], [71], [72]].

**Table 4:** ISTH Congress Report: summary of lupus anticoagulant-related studies presented at ISTH 2023 (Montreal).

• Baby et al. [24] presented their experience with the mixing study in LA testing, which was identified to reduce false LA negatives. • Several investigators provided more evidence on the utility of DOAC neutralizers for assessing LA in DOAC-treated patients [[68], [69], [70], [71], [72]]. • Bertoncin et al. [72] investigated the performance of different pairs of aPTT reagents with high and low sensitivity to phospholipids to diagnose LA.

aPTT, activated partial thromboplastin time; DOAC, direct oral anticoagulant; LA, lupus anticoagulant.

### 5.5 Future directions

Harmonization of LA testing over the last 15 years, as recommended by an earlier ISTH guideline [23], was an important initial step in reducing interlaboratory variability for LA detection. However, diagnostic errors due to preanalytical concerns remain a major problem, particularly in testing for LA on anticoagulated patients, especially DOACs. While ongoing education of clinicians is important, this issue can only be reliably overcome by instituting sample collection protocols that consider the withholding of DOACs for an appropriate time frame, if considered safe for any given patient, based on drug and renal clearance. The updated ISTH guidance [11] suggests screening LA samples with anti-Xa assays to rule out the presence of FXa inhibitors, including both LMWH/UFH and DOACs, and this information will assist in the interpretation of LA test results. However, this imposes substantial additional time burden and cost, especially for large reference laboratories, and it is not always clear which anticoagulant a patient is on, especially if the anticoagulant is not disclosed to the laboratory. Also, knowing the specific level of DOAC anti-Xa activity that interferes with LA assays is an area in need of further primary research but may be dependent on the assays used for testing.

Manufacturers have an important role to play in validating DOAC neutralizers for routine use in LA testing. Additionally, UFH neutralizers should be added to aPTT reagents marketed for use as lupus-sensitive reagents in a similar way to how they are added to dRVVT reagents. An alternative, provided by at least one manufacturer [73], is to have different options for CaCl_2_ reagents, as used to initiate coagulation in the aPTT assay, specifically with and without added heparin neutralizers—these would be applied differentially for use in LA testing vs UFH monitoring. Further development of LA tests less affected by anticoagulants (eg, Taipan- and Ecarin-based tests) could be progressed or made more widely available [19,20]. Such tests have been validated in an ISTH study for use in patients on VKA or direct FXa inhibitor DOACs [20].

## 6 Conclusion

In this narrative review, we provide an overview of LA testing, and detail some of the “good, bad, and ugly” of LA testing. It is reassuring that as problems arise (such as anticoagulant interference), some bright scientists come up with ways of neutralizing these effects to enable more accurate assessment of LA (heparin neutralizers, mixing studies for VKAs, and DOAC neutralizers). In the end, further improvements are contingent on clinicians understanding the risks imposed by testing of LA whilst patients are on anticoagulant therapy, especially DOACs, and assisting laboratories to provide more accurate test results. This requires clinical education and is often outside the control of the laboratory undertaking the testing. Mandating required information about patient anticoagulants prior to ordering of LA testing is recommended by the ISTH SSC [11], but compliance may be suboptimal in practice [25]. Accordingly, although ongoing education of clinicians is important, adopting system-based solutions for collection of LA samples after holding the anticoagulant in question for an appropriate time frame based on drug and renal clearance and providing complete anticoagulant information to the laboratory at time of test request will be needed to fully overcome these significant preanalytical concerns. For laboratories, we recommend adhering to guidance provided by the ISTH SSC on LA [11,12,20,23] or other expert groups [[13], [14], [15]] unless the laboratory has valid reasons to not do so. For example, our laboratories still use mixing studies as a strategy for minimizing the effects of anticoagulant interference, including VKAs, as once recommended [23], but no longer favored by some experts [11,12]; our reasoning is that this may represent a lesser evil than alternate options. If available, alternate methods to aPTT and dRVVT less affected by VKAs and DOACs could be considered, with the TSVT used as an LA screen and ECT as a confirmatory test in patients on VKAs or on anti-Xa agents [19,20].

Finally, there are many events outside of the laboratory control. For example, a limitation of available supply or regulatory-cleared reagents at specific locations may hinder the most effective LA test progress at individual sites.

Continued improvements in LA detection/exclusion testing over time are needed and require clinicians and laboratories to work together to adopt systematic approaches to minimize preanalytical diagnostic errors.
